# Diagnostic testing of autonomous cortisol secretion in adrenal incidentalomas

**DOI:** 10.1530/EC-20-0419

**Published:** 2020-09-10

**Authors:** Grethe Å Ueland, Thea Grinde, Paal Methlie, Oskar Kelp, Kristian Løvås, Eystein S Husebye

**Affiliations:** 1 Department of Clinical Science, University of Bergen, Bergen, Norway; 2 Department of Medicine, Haukeland University Hospital, Bergen, Norway; 3 K. G. Jebsen Center for Autoimmune Disorders, University of Bergen, Bergen, Norway; 4 Department of Medicine, Akershus University Hospital, Nordbyhagen, Norway

**Keywords:** autonomous cortisol secretion, adrenal incidentaloma, Cushing’s syndrome, DHEAS, saliva cortisol

## Abstract

**Objective:**

Autonomous cortisol secretion (ACS) is a condition with ACTH-independent cortisol overproduction from adrenal incidentalomas (AI) or adrenal hyperplasia. The hypercortisolism is often mild, and most patients lack typical clinical features of overt Cushing’s syndrome (CS). ACS is not well defined and diagnostic tests lack validation.

**Methods:**

Retrospective study of 165 patients with AI evaluated clinically and by assay of morning plasma ACTH, late-night saliva cortisol, serum DHEA sulphate (DHEAS), 24-h urine-free cortisol, and cortisol after dexamethasone suppression.

**Results:**

Patients with AI (*n* = 165) were diagnosed as non-functioning incidentalomas (NFI) (*n* = 82) or ACS (*n* = 83) according to current European guidelines. Late-night saliva cortisol discriminated poorly between NFI and ACS, showing a high rate of false-positive (23/63) and false-negative (38/69) results. The conventional low-dose dexamethasone suppression test (LDDST) did not improve the diagnostic specificity, compared with the 1 mg overnight DST. Receiver operating characteristic curve analysis of DHEAS in the two cohorts demonstrated an area under the curve of 0.76 (*P* < 0.01) with a sensitivity for ACS of 58% and a specificity of 80% using the recommended cutoff at 1.04 µmol/L (40 µg/dL).

**Conclusion:**

We here demonstrate in a large retrospective cohort of incidentaloma patients, that neither DHEAS, late-night saliva cortisol nor 24-h urine free cortisol are useful to discriminate between non-functioning adrenal incidentalomas and ACS. The conventional LDDST do not add further information compared with the 1 mg overnight DST. Alternative biomarkers are needed to improve the diagnostic workup of ACS.

## Introduction

Autonomous cortisol secretion (ACS) is usually caused by adrenal adenomas or hyperplasia and is frequently diagnosed in patients with adrenal incidentalomas (AI). AI are adrenal masses discovered on imaging undertaken for other reasons than suspicion of adrenal disease and are found in about 3% of people at 50 years of age, with the prevalence increasing to up to 10% in elderly above 70 years of age (1). Most AI are benign and non-functioning, that is, they do not cause excess hormone production. However, recent studies show that up to 30% have some degree of cortisol overproduction, depending on the diagnostic criteria applied (2).

Due to large inter- and intraindividual biological variation of cortisol secretion, most patients with ACS shows serum cortisol levels in the normal range; however, the diurnal and ultradian variation of cortisol secretion is dysregulated. Also, these patients lack the classical phenotype of overt Cushing syndrome (CS). Formerly known as subclinical CS, ACS has a much higher prevalence than overt CS, affecting up to 2% of the adult population (3). Despite the suspected link between subclinical and overt CS, evidence suggests that progression to overt CS is rare (1). Even though typical clinical findings of overt CS are lacking, patients with ACS often show one or more components of the metabolic syndrome (hypertension, type 2 diabetes mellitus, obesity and/or dyslipidemia). These findings indicate an increased risk of cardiovascular morbidity and mortality (4, 5). Moreover, ACS is associated with low bone density and osteoporosis (6, 7). The European Society of Endocrinology (ESE) and the European Network for the Study of Adrenal Tumours (ENS@T) guidelines for AI (1) have therefore recommended that all patients with AI should be assessed for ACS.

The most recent international guidelines recommend the 1 mg overnight dexamethasone suppression test (DST) to screen for ACS (1). This test is not optimal, as the DST has low diagnostic specificity (80%) yielding many false-positive test results. The use of oral estrogens and low dexamethasone bioavailability (because of poor absorption or fast metabolism) are the most important sources of error, although the latter can be overcome by parallel assay of dexamethasone in the morning sample (8). Plasma ACTH can be an add-on test to validate the ACTH-independent nature of the cortisol excess. This test has low diagnostic accuracy and precision due to analytical cross-reactivity and interference, especially in the lower range of measurements (9, 10).

Saliva cortisol (sa-cortisol) drawn at late night is commonly used in the diagnostics of overt CS, but is not found useful to reveal low graded cortisol overproduction. Sa-cortisol and the 48-h conventional low-dose (0.5 mg four times daily) dexamethasone suppression test (LDDST) are not well validated in the setting of ACS, and none of them are recommended in the ESE/ENS@T guidelines (1). Twenty-four-hour urine-free cortisol (UFC), a gold standard in the diagnosis of overt CS, is not sensitive to diagnose patients with ACS. Finally, dehydroepiandrosterone (DHEA) and its sulphated derivate (DHEAS) make up the majority of androgen precursors secreted from zona reticulata in the adrenal cortex. DHEAS has a longer half-life than p-ACTH, and the secretion is low or suppressed when ACTH is suppressed. Thus, DHEAS has the potential to be a more stable marker of adrenal hypercortisolism than p-ACTH (11, 12).

How the various diagnostic tests perform in the diagnosis of AI have been evaluated mostly in small insufficiently powered studies. Here, we checked the performance of these tests in an adequately sized patient cohort in a ‘real-life’ clinical setting.

## Patients and methods

### Study population

We retrospectively reviewed patients referred for evaluation of AI at the tertiary endocrine centers at Haukeland and Akershus University hospitals in Norway from June 2012 to August 2018. The patients in the study were collected consecutively from June 2012 to august 2016. After that, only patients with ACS were included in our biobank/registry. The participants were evaluated according to ESE and ENS@T guidelines (1). Patients diagnosed with pheochromocytomas (*n* = 3), primary aldosteronism (*n* = 1) and adrenocortical carcinomas (*n* = 1) were excluded. The study cohort consisted of the remaining patients (*n* = 165), and categorised as having either non-functioning incidentalomas (NFI, *n* = 82) or ACS (*n* = 83). The ESE guidelines recognise three categories of patients based on post DST cortisol levels. S-cortisol below 50 nmol/l is named NFI, between 50 and 139 nmol/L is named possible ACS and 140 nmol/L or above is ACS. We have defined ACS here as a s-cortisol value above 50 nmol/L after the 1 mg overnight DST (positive test). This because we have excluded the most common reasons for a false DST, namely patients with low dexamethasone bioavailability < 3.3 nmol/L (*n* = 5) (8), and patients using oral estrogens. In addition, all patients had a p-ACTH analysed and were also asked to obtain two late-night saliva samples for sa-cortisol. If the DST was positive, the patients underwent a 24 h UFC measurement and a LDDST. One patient with adrenal incidentaloma was diagnosed with pituitary CS and excluded.

### Assay of hormones

S-cortisol were assayed by an in-house LCMS/MS method, previously described in detail (13). The reference range was 120–600 nmol/L for samples drawn before 10:00 h (14). The assay precision was 4.5–7.4% relative standard deviations (RSD), and the accuracy ranged from 97 to 101% (13). DHEAS was analysed by chemiluminescent immunoassay (CLIA) using Siemens Immulite 2000 XPi. Age and gender-specific reference ranges for the method are given in Table 1. Intraindividual biological variation was estimated to be 6.35% and the analytical precision was RSD 9% at concentration level 12 µmol/L (15). DHEAS was included in the routine diagnostic workup of patients with AI from January 2015. Thus, some patients included before this date lack data on DHEAS. Saliva was collected by an oral swab (Salivette, Sarstedt, Germany) held in the mouth for about 2 min at 23:00 h. Patients were instructed not to eat, brush their teeth, smoke, chew tobacco or use snuff for at least 1 h before sample taking. The samples were analysed using LCMS/MS. Positive tests were defined as sa-cortisol > 2.8 nmol/L, which is the well-validated cutoff level used for diagnosing overt CS at our laboratory. The analytical precision was RSD 9% at concentration 3.1 nmol/L (16). P-ACTH samples were collected between 08:00 and 09:00 h, analysed by CLIA using Siemens Immulite 2000 XPi. Intraindividual biological variation was estimated to be 15%. The lower limit of quantification was 1.1 pmol/L and the reference range was 2.0–11.6 pmol/L in samples drawn before 10:00 h. The analytical precision was RSD 8 at a concentration of 4 pmol/L (17). Urine was collected over a period of 24 h, and analysed using LCMS/MS. The concentration of the excreted-free cortisol was calculated relative to the amount of urine produced. A normal test was defined as a cortisol level below 165 nmol/24 h, a well-validated cutoff level used to diagnose overt CS at this method. Analytical precision was RSD 10% at a 140 nmol/L concentration (18).

**Table 1:** Age- and sex-adjusted reference range for DHEAS (μmol/L).

Age, years	Sex	Lower limit (μmol/L)	Upper limit (μmol/L)
18–29	Female	1.5	11.6
	Male	2.2	13.5
30–39	Female	1.6	11.0
	Male	2.1	11.6
40–49	Female	0.7	7.8
	Male	1.5	9.7
>50	Female	0.4	4.9
50–60	Male	1.1	11.5
>60	Male	0.4	5.4

### Statistics

Categorical data are given as number (%) and continuous data as median (range). Since the data was not normally distributed, non-parametric statistics were applied. A significance level of 0.05% was chosen. The Mann–Whitney *U*-test was used to compare groups. Spearman correlation was used to evaluate the degree of correlation when appropriate. A receiver operating characteristic (ROC) curve was made for DHEAS and sa-cortisol to evaluate the diagnostic performance, and for DHEAS also to evaluate the proposed cutoff of 1.04 µmol/L (40 µg/dL) in the diagnostics of ACS (11).

### Ethics

The study was approved by the local ethics committee (REK) and all participants signed an informed consent form (REK no: 2011/1810 and 2014/2170).

## Results

### Patient characteristics

One hundred and sixty-five patients were included in the study; 82 had NFI, and 83 had ACS (Table 2). Overall, patients with ACS had more cardiovascular risk factors than those with NFI. They were more often treated with anti-hypertensive medication and lipid-lowering drugs, and had a higher prevalence of type 2 diabetes mellitus and osteoporosis despite being less obese (Table 2).

**Table 2:** Characteristics of the subjects in the study.

	NFI (*n* = 82)	ACS (*n* = 83)
Women, *n* (%)	48 (56.1)	58 (69.9)
Age, median, years (range)	68.5 (33–82)	65 (29–86)
BMI, median, kg/m^2^ (range)	28.6 (18.3–42.6)	26.1 (16.4–45.2)
Hypertension, *n* (%)^a^	42 (50.9)	54 (64.8)
Diabetes type II, *n* (%)^a^	7 (8.5)	14 (16.7)
HbA1c, median, % (range)	5.7 (5–9)	5.8 (5–9)
Treatment for dyslipidemia, *n* (%)^a^	16 (19.5)	18 (21.7)
Osteoporosis, *n* (%)^a^	7 (8.5)	15 (18.1)
Creatinine, median, µmol/L (range)	75 (40–138)	72 (39–971)
Smokers, *n* (%)^a^	31 (37.8)	30 (36.1)
Unilateral AI, *n* (%)	66 (81.0)	66 (79.2)
Size of unilateral AI, median, mm (range)	22 (9–70)	24 (10–51)
Bilateral AI, *n* (%)	16 (19.0)	17 (20.8)
Size of largest AI if bilateral, median, mm (range)	28 (13–45)	33 (21–42)

Categorical data are given as number and percent, continuous data as median and range. In the case of bilateral AI, only the largest of the two lesions was included when calculating median size and range.

^a^Significant difference between the NFI and the ACS group. *P* < 0.05.

### Diagnostic testing of the cohort

The results of the diagnostic tests performed in the two groups of patients are summarised in Table 3. ACTH was assayed in plasma, and measurements were available in 74 of the 83 ACS patients, and in 61 of the 82 NFI patients. The median p-ACTH was 1.65 pmol/L (range, <1.1-5.5 pmol/L) and 3.9 pmol/L (range <1.1–40.2 pmol/L) in the ACS and NFI groups, respectively (*P* < 0.05). The median s-cortisol after DST was 84 nmol/L (range 51–486) in the ACS group, and 31 nmol/L (range 12–50) in the NFI group.

**Table 3:** Results of diagnostic testing.

	NFI	ACS
Basal cortisol, median, nmol/L (range)^a^	320 (110–629)	411 (130–985)
Morning ACTH, median, pmol/L (range)^a^	3.9 (1.1–40.2)	1.65 (1.1–5.5)
Cortisol after 1 mg DST, median, nmol/L (range)^a^	31.0 (12–50)	84 (51–486)
Late-night saliva cortisol, median, nmol/L (range)^a^	0.9 (0.1–8.2)	1.75 (0.4–45)
DHEAS, median, µmol/L, (range)^a^	1.85 (0.4–10.5)	0.95 (0.4–3.8)
UFC, median, nmol/24 h (range)		72 (2–321)
LDDST, median, nmol/L (range)		89 (58–517)

Continuous data as median and range. Lower limit of quantification for p-ACTH, s-cortisol after DST and s-DHEAS was put as registered value for all patients who tested below the limit (<1.1 pmol/L, <28 nmol/L and <0.4 µmol/L for p-ACTH, s-cortisol after DST and s-DHEAS, respectively).

^a^Significant difference between the NFI and the ACS group.

#### Saliva cortisol

**Figure 1:**
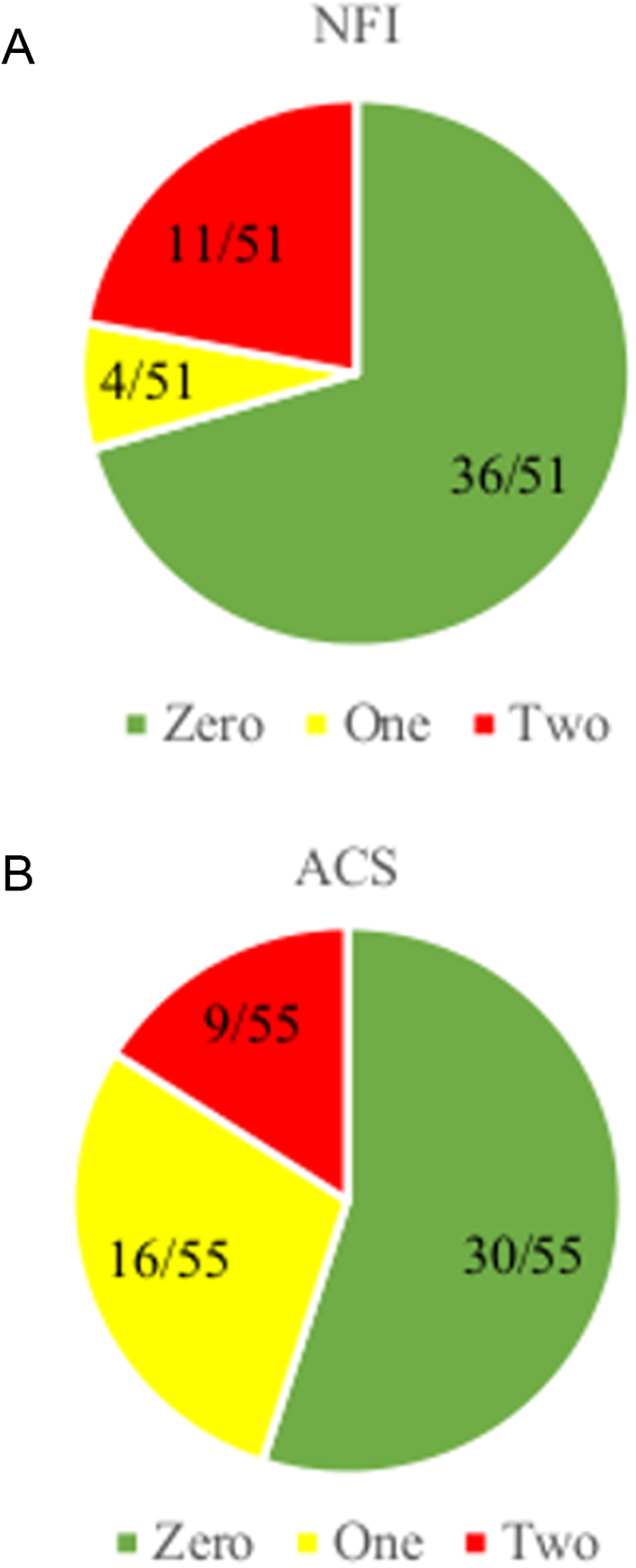
Proportion of patients with zero (green), one (yellow) and two (red) positive saliva samples, respectively, in the NFI group and the ACS group. Only patients who had two saliva samples analysed were included in this graph.

In the ACS group, we had valid saliva data for 69/83 patients, 14/69 patients had one saliva sample performed and 55/69 patients had two. In the NFI group, 63/82 patients had valid saliva data, one saliva sample was obtained in 12/63 patients and 51/63 patients delivered two samples. The median sa-cortisol level was higher in the ACS than in the NFI group (1.75 nmol/L (0.4–45) vs 0.9 nmol/L (0.1–8.2); *P* < 0.05) even though both medians were below the cutoff for a normal test (2.8 nmol/L). Thirty-eight of sixty-nine patients in the ACS group and 40/63 in the NFI group had normal saliva samples. However, in patients who delivered two saliva samples, 16/55 in the ACS group and 4/51 with NFI had one positive and one negative measurement (Fig. 1). In the ACS group, 30/55 patients with two samples tested negative in both (two false-negative tests), while 11/51 in the NFI group had two positive tests (two false-positive tests) (Fig. 1). There were no significant differences in the proportions of positive and negative saliva test results between patients with post DST cortisol above and below 140 nmol/L. A receiver operating characteristic (ROC) curve for late-night saliva cortisol as a marker of ACS showed a low area under the curve (AUC) of 0.65 (CI 0.58–0.72, *P* < 0.05), and there were not possible to derive a better cutoff level than 2.8 nmol/L, suitable to differentiate between ACS and NFI.

#### Dehydroepiandrosterone sulphate

**Figure 2:**
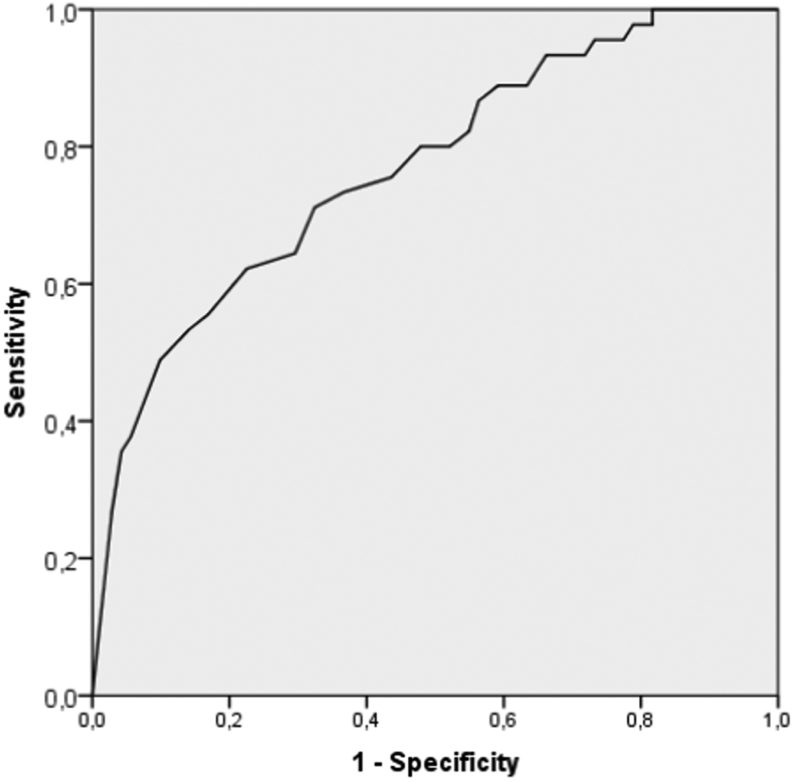
ROC curve of DHEAS.

In the ACS group, we had valid DHEAS data for 58/83 patients, and in the NFI group 54/82 patients. The median value of DHEAS was 0.95 µmol/L (range <0.4–3.8 µmol/L) in the ACS group, compared to 1.85 µmol/L (range <0.4–10.5, *P* < 0.001) in the NFI group. A proposed cutoff for DHEAS in the diagnostics of ACS is 1.04 µmol/L (40 µg/dL), using the exact same method as our laboratory (11). In the ACS group, 32/38 patients had DHEAS measurements equal to or below this cutoff compared to 11/54 patients in the NFI group. There were no significant difference between median post DST cortisol (33 nmol/L) in patients with NFI and low DHEAS, compared to those with NFI and DHEAS above 1.04 µmol/L (30 nmol/L). There were no significant differences in the proportions of patients with DHEAS above and below 1.04 µmol/L between patients with post DST cortisol above and below 140 nmol/L. Figure 2 demonstrates a ROC curve for DHEAS as a marker for ACS with an area under the curve (AUC) of 0.76 (CI 0.68–0.85, *P* < 0.05). Applied to our cohort, the cutoff of 1.04 µmol/L (40 µg/dL) yields a sensitivity of 58% and a specificity 80% for diagnosing ACS.

Dennedy et al. have suggested to calculate a ratio derived by dividing DHEAS by the lower limit of the sex- and age-specific reference range for the analysis. We used the recommended lower limits shown in Table 1 for the ratio calculations (12). We found a median DHEAS ratio significantly lower in the ACS group (1.75, CI 1.0–9.5), compared to the NFI group (3.0, CI 0.26–26.5, *P* < 0.05). Calculating a ROC curve for the use of DHEAS-ratio in the diagnostics of ACS gave an AUC of 0.69 (CI 0.58–0.79, *P* < 0.01).

**Figure 3:**
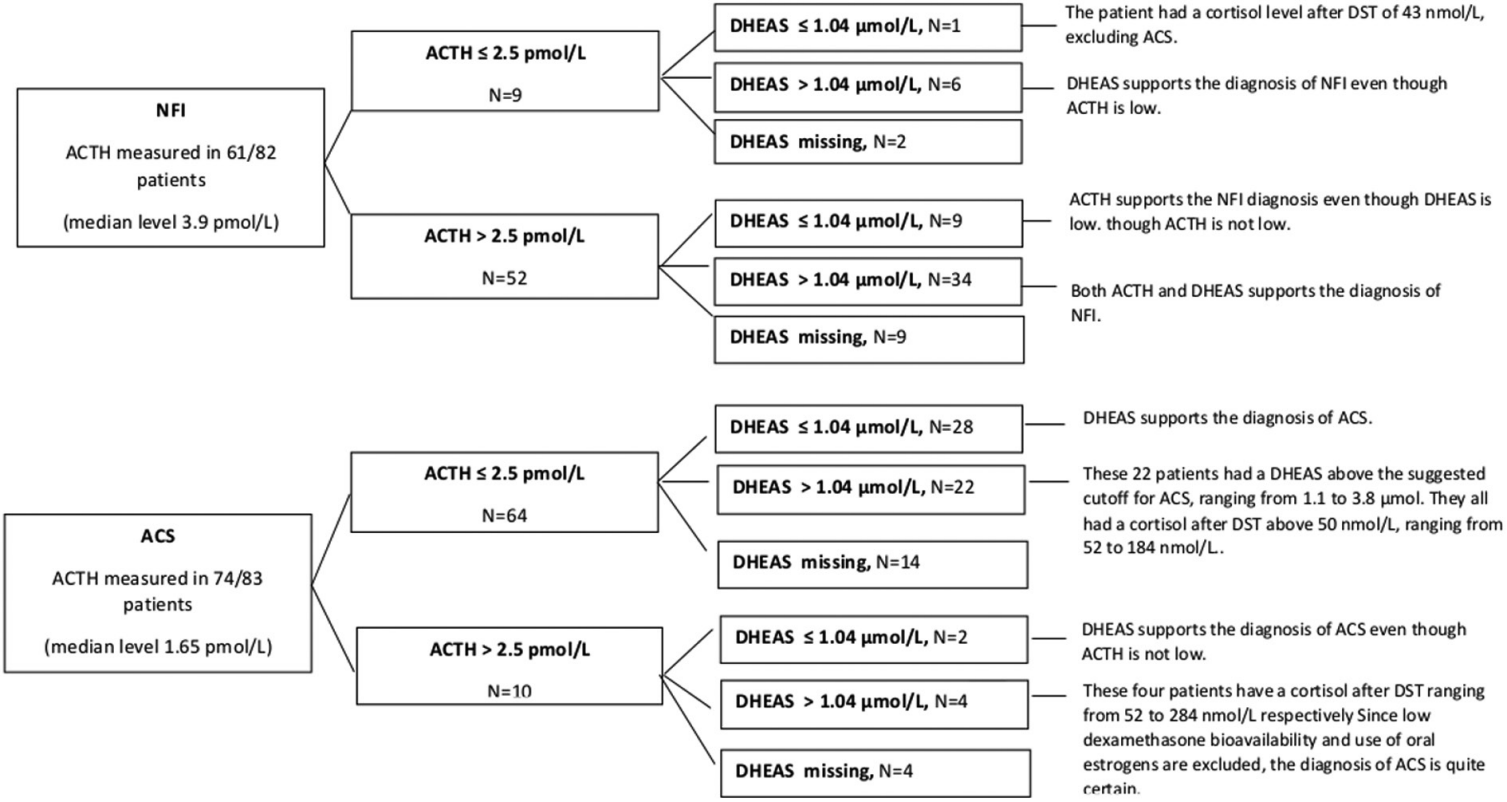
ACTH and DHEAS levels in patients with NFI and with ACS.

Figure 3 shows that there is a poor agreement between the occurrence of low DHEAS and low p-ACTH in both patient cohorts. The median s-creatinine level in the whole study cohort was 73 µmol/L (39–971). In the ACS group, the median level was 72 µmol/L (39–971), and in the NFI group 75 µmol/L (40–138). There were no significant correlations between DHEAS and s-creatinine, whether the cohort was examined in total (Spearman rho 0.188, *P* = 0.12) or reviewed as two separate groups

#### Dexamethazone suppression testing

The median level of s-cortisol after the LDDST in the ACS group was 89 nmol/L (51–517, Table 3). A scatterplot comparing s-cortisol results after the overnight DST and the LDDST revealed a Spearman correlation coefficient of 0.812 (*P* < 0.001).

#### Urinary-free cortisol

UFC was assayed once (*n* = 49/58) or twice (*n* = 29/58) in the cohort of ACS. Seven patients had one or more positive test results. Two patients tested positive in both samplings, while four tested positive in one sample. One delivered a single positive urine. The two patients with two positive UFC were from the category of patients with post-DST-cortisol above 140 nmol/L.

## Discussion

Low DHEAS levels have been postulated as a promising diagnostic biomarker of ACS (11, 12, 19). Production is stimulated by ACTH, but DHEAS has a longer half-life than cortisol, which potentially makes it a more stable and reliable marker of cortisol excess. However, we find that DHEAS cannot be trusted as a biomarker of ACS. Neither a previously suggested cutoff (11), nor ratio (12) derived from studies using the exact same DHEAS assay as we, were useful to discriminate between ACS and NFI. ROC analysis revealed a fairly good AUC of 0.76 for ACS. Although the diagnostic performance of DHEAS in our hands was similar to the original study by Yener, sensitivity of 58% and specificity of 80% is not convincing. Furthermore, our ROC-curve did not indicate another cutoff for DHEAS that would have improved the separation of ACS patients from healthy subjects.

The use of p-ACTH in the diagnostics of ACS is challenged by falsely high values in samples with low p-ACTH in some cases (9, 10). There are also challenges related to the sampling as p-ACTH is an unstable analyte needing immediate freezing.

When evaluating the use of DHEAS in the diagnostics of ACS there are several challenges. The current diagnostic criteria of ACS may be based on insufficient and uncertain data. ACS patients overlap clinically, radiologically as well as biochemically with the general population. Without a clearly defined group of patient with ACS identified by optimal gold-standard diagnostic test, it is likely that the conclusions are influenced by patient selection. The use of different inclusion criteria and cutoffs in different studies adds to the confusion. The optimal study design should include endpoints defining improvement of comorbid conditions (diabetes mellitus, hypertension, obesity) or mortality data after surgery. Furthermore, many patients with ACS are quite old, an age group expected to have physiologically low DHEAS levels.

DHEAS as a sulphate conjugate is excreted through the kidneys, and higher levels may be expected in patients with kidney failure. We found no difference between s-creatinine in the two groups, and there were no correlation between s-creatinine and DHEAS in the whole cohort, or when each patients group were reviewed separately. Thus, it is unlikely that our findings are confounded by kidney function.

The activity of enzymes involved in the sulphation of DHEA to DHEAS may vary between individuals and increase variability; thus DHEA could perhaps be a substitute. However, assay of DHEA with its shorter half-life and circadian variation is not straight forward.

Furthermore, the manufacturer does not state any known interference issue and we are not aware of any such reports, other than heterophilic autoantibodies, concerning the Immulite 2000 DHEAS analysis. This may be expected because DHEAS are present in blood at levels orders of magnitude higher than other steroid hormones.

Sa-cortisol has high diagnostic accuracy (sensitivity 92–100%, specificity of 93–100%) in the diagnosis of overt CS (20). However, the method is not validated for low-grade ACS. Our results show that the test is useless to differentiate between ACS and NFI. The median value in both groups was below the proposed cutoff level, and the majority of those with ACS were, based on saliva measurements, categorised as healthy. Only 9/55 of the ACS patients tested positive in both saliva samples. Another problem with sa-cortisol is a large number of false positives in the NFI group (15/51 had at least one positive sample). Although these numbers are discouraging, one might speculate whether false positives in the NFI group could represent a low degree of hypercortisolism in some cases. Ceccato *et al.* found that the circadian cortisol rhythm was not impaired using serial sa-cortisol samples and calculating AUCs. The cortisol exposure was only increased in the morning in patients with ACS (21). This corroborates our findings that late-night sa-cortisol is unsuitable as a diagnostic tool in ACS. Our attempt to define an alternative cutoff level for sa-cortisol suitable to distinguish between NFI and ACS, did not reveal a cutoff level with a better sensitivity and specificity than 2.8 nmol/L used to diagnose overt CS.

The LDDST has mainly been used in a further assessment of patients with positive overnight DST, when there are discrepancies between the overnight DST and other diagnostic tests. After excluding patients with low dexamethasone bioavailability, we found a very strong correlation between the overnight DST and LDDST. The LDDST was not able to exclude ACS in any patients diagnosed by DST. Thus, we found no support for the continued use of LDDST in the diagnostics of ACS.

The UFC-test has been considered the gold standard in the diagnostics of overt CS since the 1970s (22), but have shown poor sensitivity for low-grade hypercortisolism (23). Our study supports this finding as only seven of the 83 patients in the ACS group had one or more positive UFC.

Despite the fact that we here report findings in a sizeable cohort of subjects, the retrospective nature of the study is an important limitation. Furthermore, a significant amount of missing data for both DHEAS, sa-cortisol and UFC and p-ACTH is an important shortcoming. Also, two salivary cortisol samples were only available for 55/83 ACS and 51/82 NFI patients – which given the well-recognised limitations of a single salivary cortisol measurements, is a weakness worth mentioning.

In conclusion, we recognise that the conventional approach using spot measurements, dynamic testing and daily hormone excretion are far from suitable to accurately diagnose ACS. DHEAS was not found to be an adequate replacement for p-ACTH in the diagnostics of ACS. Late-night sa-cortisol was not useful to distinguish between ACS and NFI, and the LDDST did not increase the diagnostic specificity compared to the 1 mg overnight DST. The UFC had insufficient sensitivity for detecting patients with ACS. Thus, for the time being, morning ACTH and the 1 mg overnight DST are the tests of choice.

## Declaration of interest

The authors declare that there is no conflict of interest that could be perceived as prejudicing the impartiality of the research reported.

## Funding

This work was supported by a grant from Stiftelsen Kristina Gerhard Jebsen and The regional Health Authorities of Western Norway.
